# Pressurized Intraperitoneal Aerosol Chemotherapy (PIPAC) with Low-Dose Cisplatin and Doxorubicin in Gastric Peritoneal Metastasis

**DOI:** 10.1007/s11605-015-2995-9

**Published:** 2015-10-28

**Authors:** Giorgi Nadiradze, Urs Giger-Pabst, Juergen Zieren, Dirk Strumberg, Wiebke Solass, Marc-André Reymond

**Affiliations:** Department of Surgery, University of Magdeburg, Magdeburg, Germany; Department of Surgery, Ruhr University Bochum, Bochum, Germany; Department of Internal Medicine, Oncology and Haematology, Ruhr University Bochum, Bochum, Germany; Institute of Pathology, Medical School Hanover, Hanover, Germany; Marienhospital Herne, Ruhr University Bochum, Hölkeskampring 40, 44625 Herne, Germany

**Keywords:** Gastric cancer, Peritoneal metastasis, Pressurized intraperitoneal aerosol chemotherapy, Intraperitoneal chemotherapy, Cisplatin, Doxorubicin

## Abstract

**Background:**

Pressurized intraperitoneal aerosol chemotherapy (PIPAC) is a novel technique of intraperitoneal chemotherapy. First results obtained with PIPAC in patients with advanced peritoneal metastasis (PM) from gastric cancer (GC) are presented.

**Methods:**

Retrospective analysis: Sixty PIPAC were applied in 24 consecutive patients with PM from GC. 67 % patients had previous surgery, and 79 % previous platinum-based systemic chemotherapy. Mean Peritoneal Carcinomatosis Index (PCI) of 16 ± 10 and 18/24 patients had signet-ring GC. Cisplatin 7.5 mg/m^2^ and doxorubicin 1.5 mg/m^2^ were given for 30 min at 37 °C and 12 mmHg at 6 week intervals. Outcome criteria were survival, adverse events, and histological tumor response.

**Results:**

Median follow-up was 248 days (range 105–748), and median survival time was 15.4 months. Seventeen patients had repeated PIPAC, and objective tumor response was observed in 12 (12/24 = 50 %): no vital tumor cells = 6, major pathological response = 6, minor response = 3. Postoperative adverse events > CTCAE 2 were observed in 9 patients (9/24, 37.5 %). In 3/17 patients, a later PIPAC could not be performed due to non-access. Two patients (ECOG 3 and 4) died in the hospital due to disease progression.

**Conclusion:**

PIPAC with low-dose cisplatin and doxorubicin was safe and induced objective tumor regression in selected patients with PM from recurrent, platinum-resistant GC. First survival data are encouraging and justify further clinical studies in this indication.

**Electronic supplementary material:**

The online version of this article (doi:10.1007/s11605-015-2995-9) contains supplementary material, which is available to authorized users.

## Introduction

Gastric cancer is the second most common cause of death from cancer worldwide.^1^ In most patients with gastric cancer, therapy is palliative and systemic chemotherapy including 5-FU/platinum combinations, with or without an anthracycline, as well as irinotecan and docetaxel-containing combinations is the current standard of care.^2^ Peritoneal carcinomatosis develops in more than half of patients.^3^ These patients have a poor prognosis with a median survival of 3–5 months without treatment.^4^,^5^ No large-scale comparative studies document the efficacy of systemic chemotherapy in gastric peritoneal metastasis, but it appears to be modest.^6^

Pressurized intraperitoneal aerosol chemotherapy (PIPAC) is a novel technique delivering drugs into the abdominal cavity as a aerosol under pressure. Taking advantage of physical laws, it is generating an artificial pressure gradient enhancing tissue uptake and distributing drugs homogeneously within the closed and expanded abdominal cavity. Prior experimental work has documented the favorable effects of applying pressure into the peritoneal cavity,^7^–^9^ by counteracting the elevated intratumoral interstitial fluid pressure^10^ and enhancing drug uptake by convection. PIPAC is a short laparoscopic procedure and can be repeated. Only approved chemotherapeutic drugs were applied so far so that a cytotoxic activity is expected after PIPAC application.

In an ex vivo model of human peritoneal metastasis, PIPAC resulted in a higher local concentration of a small molecule (Dbait) compared to intraperitoneal lavage and in a deeper tissue penetration.^11^ In the abdomen of pigs, PIPAC allowed a homogeneous distribution of a staining agent as compared to closed liquid circulation.^12^ Based on these encouraging data, PIPAC was first applied in November 2011 in 3 end-stage patients with peritoneal metastasis.^13^ Courses of PIPAC containing cisplatin 7.5 mg/m^2^ and doxorubicin 1.5 mg/m^2^ (about 10 % of an usual systemic dose) at 12 mmHg and 37 °C for 30 min were applied q 28–42 days. The application was well tolerated and superior pharmacological properties confirmed. Intratumoral concentration of doxorubicin was much higher (up to 2 × 10^2^) than reported after HIPEC, with 10 % of the usual dose. Systemic drug concentration remained low, and PIPAC caused almost no hepatic and renal toxicity.^14^ No local complication was recorded. However, a strong increase of C-reactive protein was observed as a sign of the chemical peritonitis. Regression of peritoneal nodules was observed in all three patients, and a patient survived 2 years after first PIPAC.

In a retrospective case series of 18 women with recurrent ovarian cancer, repeated courses q 28–42 days of PIPAC with the same regimen were safe and effective when applied without concomitant cytoreductive surgery. These results indicated a PIPAC activity in women with recurrent, platinum-resistant ovarian cancer.^15^ Recently, a regulatory prospective phase-2 trial with low-dose doxorubicin and cisplatin applied as a pressurized aerosol in recurrent, platinum-resistant ovarian cancer showed a clinical benefit rate (CBR) of 62 % and an objective histological regression rate of 76 % coupled with a low incidence of severe adverse events: 15 % CTCAE 3, no CTCAE 4 and 5.^16^ Thus, PIPAC might become a new and promising drug delivery technique for ovarian cancer patients in the recurrent and perhaps also in the adjuvant setting.^17^,^18^

We now report about our first observations with low-dose PIPAC application in patients with gastric peritoneal metastasis.

## Methods

In fall 2011, we opened a PIPAC program for patients diagnosed with advanced, therapy-resistant gastric peritoneal metastasis. Therapy was conducted in accordance with the Helsinki’s declaration. All patients gave their informed consent. The Ethics Committees of the Ruhr University Bochum, Germany expressed no objection. Access to this off-label use program was limited to patients who had a life-threatening disease, including some patients with advanced disease in reduced general condition (ECOG 3 and 4) and with large amount of ascites.

Prior to therapy, each patient was evaluated by the multidisciplinary tumor board at the Marien Hospital Herne, Ruhr-University Bochum, Germany. There were no specific inclusion or exclusion criteria, and therapeutic indication was individual. All patients had histologically verified peritoneal metastasis of gastric origin, no option for complete cytoreductive surgery (CRS) and hyperthermic intraperitoneal chemotherapy (HIPEC) because of poor general condition, signet cell histology, advanced PCI, and/or diffuse small bowel involvement. Most of them had previous palliative systemic chemotherapy. A few patients were medically unfit for systemic palliative chemotherapy or refused to receive it. Patients with other metastatic localization were not treated (with the exception of pleural effusion). Reduced general condition (Karnofsky ≤ 60 %), therapy-resistant ascites, and partial small bowel obstruction were not considered as exclusion criteria.

All interventions were performed under general anesthesia. After insufflation of a 12 mmHg capnoperitoneum (with open access or Veres needle), two trocars (5 and 12 mm, Kii®, Applied Medical, Düsseldorf, Germany) were inserted into the abdominal wall. Ascites were removed. Extent of peritoneal carcinomatosis was determined.^19^ Peritoneal biopsies were taken in all 4 quadrants, and a centimetric local peritonectomy was performed to improve accuracy of histopathology, in particular when biopsies remained negative. A micropump (MIP®, Capnomed, Villingendorf, Germany) was connected to an intravenous high-pressure injector (Arterion Mark 7®, Medrad, Germany) and inserted into the abdomen. Tightness of the abdomen was documented via a zero flow of CO_2_. The procedure was performed in a room equipped with laminar air flow. A pressurized aerosol containing doxorubicin at a dose of 1.5 mg/m^2^ body surface in a 50 ml NaCl 0.9 % followed by cisplatin at a dose of 7.5 mg/m^2^ in a 150-ml NaCl 0.9 % was applied. Flow was 30 ml/min, and upstream pressure was 200 psi. Injection was remote-controlled and nobody remained in the room during application. The therapeutic aerosol was maintained at 12 mmHg for 30 min at 37 °C. Then, it was released safely via a Closed Aerosol Waste System (CAWS). Trocars were retracted and laparoscopy ended. No drainage was applied.

Follow-up was obtained by telephone calls until November 21st, 2013 or until death. All data were documented according to our institutional rules, including electronic archiving and video recording of the procedures. Histological tumor response was assessed by an independent anatomopathologist. Adverse events were graded according to the Common Terminology Criteria for Adverse Events (CTCAE). Analysis was retrospective. Survival was modelled in a Kaplan–Meier curve. We used SPSS for Windows (v.20.0, SPSS Inc., Chicago, IL) for analysis.

## Results

A total of 25 consecutive patients were scheduled for PIPAC. In one patient, access to the abdominal cavity was not possible due to extensive adhesions (*n* = 1/25; 4 % primary non-access). Twenty-four patients (M/F = 12:12) with a mean age of 56 (±13) years received ≥1 PIPAC and are object of further analysis. Patient’s characteristics and preoperative details are summarized in Table 1. Most tumors were classified as diffuse or signet-ring cancers (18/24, 75 %), and peritoneal metastasis was advanced (mean PCI of 16 ± 10). Three patients had malignant pleural effusion, and liver metastasis was diagnosed at PIPAC #2 in a further patient so that 4 patients had extraperitoneal metastasis. Five patients (21 %) were in reduced general condition (ECOG > 2); in 3 patients, systemic chemotherapy was contraindicated. Most patients were pre-treated: 19/24 (79 %) had previous (radio)chemotherapy, and 11 patients (46 %) were in the 3rd line or 4th line situation. Fourteen patients (58 %) had previous gastrectomy; a further patient had intestinal bypass surgery. Eight patients (33 %) received PIPAC in combination with systemic chemotherapy.

**Table 1:** Characteristics of 24 patients with PC from gastric origin undergoing pressurized intraperitoneal aerosol chemotherapy (PIPAC)

Variable	Value
Number of patients	24
Sex (M/F)	12:12
Age, years (±SD)	56 (±13)
Histology (Lauren classification)	
Diffuse/signet ring	18
Mixed	1
Intestinal	5
Extraperitoneal metastasis	4 (17 %)
Pleura	3
Liver	1
Peritoneal Carcinomatosis Index (mean ± SD)	16 (±10)
Karnofsky Index before first PIPAC (mean ± SD)	78 (±22 %)
Previous organ surgery	15/24 (63 %)
Previous (radio)chemotherapy	19 (79 %)
≥3 lines	4
2 lines	7
1 line	8
Contraindication for chemotherapy	3
Patient refusal	2
Simultaneous chemotherapy	8 (33 %)

A total of 60 successful PIPAC procedures were performed in the 24 patients. Seventeen patients (71 %) had repeated PIPAC (2 patients: 5 PIPAC, 5 patients: 4 PIPAC, 3 patients: 3 PIPAC, 7 patients: 2 PIPAC. In 3 patients, non-access of the abdomen because of adhesions prevented repeated PIPAC application (at 2nd, 3rd, and 6th intended PIPAC session). Mean operating time was 91 ± 34 min. Three intraoperative complications were noted (3/60, 5 %), including two bowel access lesions that were repaired (CTCAE 2). One severe allergic reaction to metamizol was controlled with corticoids and volume therapy.

**Fig. 1:**
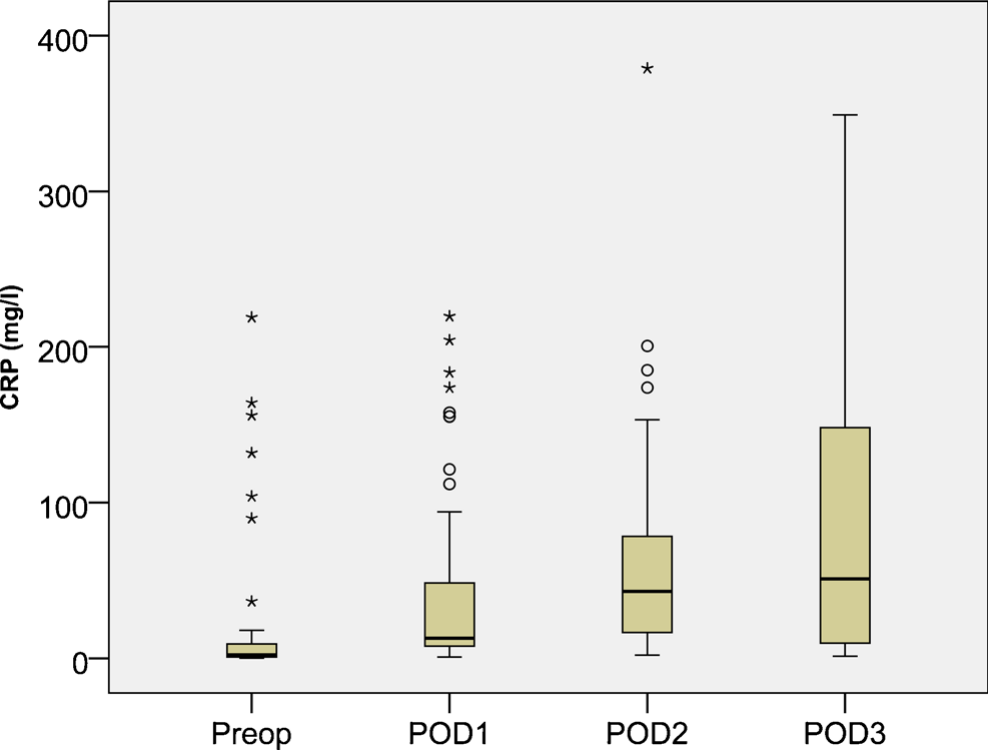
PIPAC is well tolerated. Although the dose applied is only 10 % of a usual systemic dose, patients develop a postoperative inflammatory syndrome with elevated C-reactive protein, probably explained by a chemical peritonitis. However, acute and cumulative local toxicities of PIPAC are well controlled and no bowel perforation and no gastrointestinal side effects > CTCAE grade 2 were observed

PIPAC was well tolerated. Adverse events are summarized in Table 2. Abdominal pain CTCAE ≤2 was noted in 6/24 patients (25 %). Elevated postoperative serum C-reactive protein was observed in most patients (16/24, 67 %) as a sign of the chemical peritonitis (Fig. 1). A patient with biliary stent developed postoperative cholangitis, another developed erythema at an abdominal port site. In one case, upper gastrointestinal bleeding was suspected but not confirmed. No procedure-related mortality was noted. Two patients in reduced general condition died in the hospital, one of them (ECOG 4, ASA4, therapy-resistant ascites, and pre-existing renal failure) because of lung edema after 1st PIPAC, and the other patient (ECOG 3) because of progressive small bowel obstruction after 2nd PIPAC. In the first case, cardiopulmonary decompensation was explained by ascites removal in a critically ill patient; in the second case, bowel obstruction was explained by disease progression and bowel invasion. A direct causal relationship with PIPAC (laparoscopy and application of low-dose cisplatin and doxorubicin) was considered to be unlikely.

**Table 2:** Adverse events

Patient	Operation	Response	CTCAE grading	Adverse event
1	2 × PIPAC, small bowel resection	CR	1	Abdominal pain
2	1 × PIPAC	N/A	1	CRP
3	4 × PIPAC	CR	1	CRP
4	1× PIPAC, ileostomy	N/A	3	CRP, Cholangitis
5	4 × PIPAC	PR	3	Hepatotoxicity, abdominal pain
6	2× PIPAC	SD	1	CRP
7	4× PIPAC	PD	1	CRP
8	1× PIPAC	N/A	1	Abdominal pain
9	5× PIPAC	CR	1	Abdominal pain, CRP
10	5 × PIPAC, adhesiolysis, incisional hernia repair	CR	1	Hepatotoxicity, CRP
11	4× PIPAC	PR	4	Allergy, myolysis
12	3× PIPAC, small bowel resection	PR	3	Hepatotoxicity, abdominal pain
13	3× PIPAC	CR	1	Abdominal pain, CRP
14	1× PIPAC	N/A	1	CRP
15	2× PIPAC	PR	1	CRP
16	2× PIPAC	PR	1	N/V, CRP
17	3× PIPAC	SD	1	Renal toxicity, CRP
18	2× PIPAC, gastrectomy	CR	1	CRP
19	2× PIPAC	PR	5	CRP, Hepatotoxicity, progressive SBO, death
20	1× PIPAC	N/A	3	CRP, Hepatotoxicity
21	2× PIPAC	PD	3	Hepatotoxicity
22	1× PIPAC	N/A	5	Ascites decompensation, death
23	5× PIPAC	SD	3	Hepatotoxicity, CRP, 2 × Access lesion
24	1× PIPAC	N/A	1	Access site extravasation

*PIPAC* pressurized intraperitoneal aerosol chemotherapy, *CRS* cytoreductive surgery, *CTCAE* Common Terminology Criteria for Adverse Events Version 4.0, *CR* complete remission, *PR* partial remission, *SD* stable disease, *PD* progressive disease, *CRP* C-reactive protein, *RT* renal toxicity, *N/V* nausea–vomiting, *SBO* small bowel obstruction

**Fig. 2:**
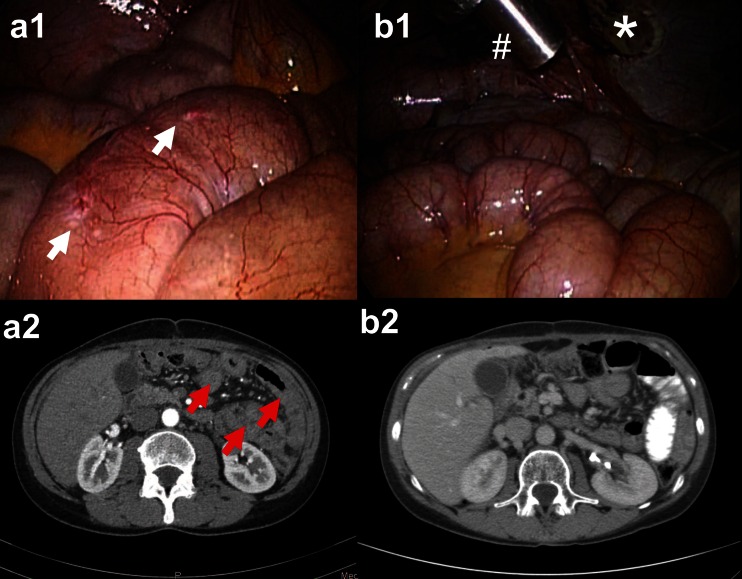
Fifty-one years old female patient after R1 gastrectomy, postoperative chemotherapy (FLOT), and radiochemotherapy (5-FU) for GC, intestinal type, pT3 pN2 pM1 (per). Videolaparoscopy (*a1*) and CT scan (*a2*) at PIPAC#1 showing multiple small bowel involvement (*white arrows*) and radiological diffuse small bowel thickening (*red arrows*). At PIPAC # 4, videolaparoscopy shows a complete macroscopic response (*b1*) and CT a complete radiological response according to RECIST 1.1 criteria (*b2*). *Number sign*: micropump placed into the abdomen during laparoscopy. *Asterisk*: local peritonectomy scar. Multiple biopsies confirm major remission with extensive fibrosis and isolated vital tumor cells. Patient was alive 148 days after 1^st^ PIPAC with a KI of 90 %

Out of 24, a total of 17 patients having received ≥2 PIPAC cycles were eligible for tumor response assessment. In 6 patients, no vital tumor cells were found (complete histological remission); in 6 further patients, partial regression was documented and in three stable disease. Thus, in total, histological response was confirmed in 50 % (12/24) patients. Examples of tumor regression are shown in Fig. 2 and Suppl. Material 1.

**Fig. 3:**
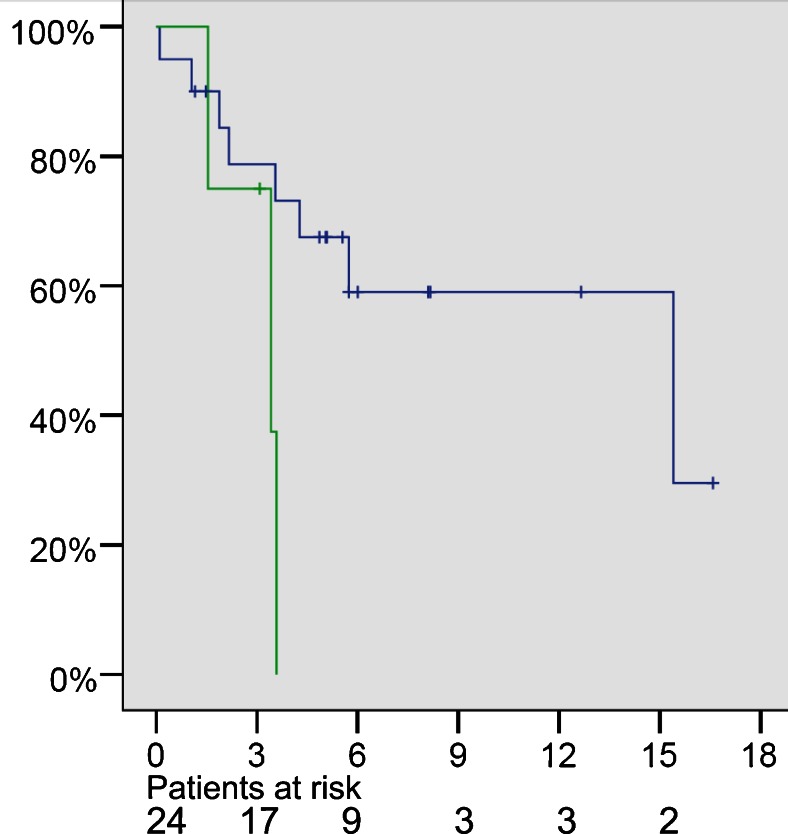
Kaplan-Meier survival curve of 24 consecutive patients after PIPAC salvage therapy with cisplatin and doxorubicin. *x*-axis: survival in months; *y*-axis: cumulative survival. *Green line*: patients with peritoneal carcinomatosis (PC) plus other metastases. *Blue line*: patients with PC without other metastases

Median follow-up was 248 days (range 105–748). At the end of follow-up, 13/24 patients were alive. Median survival overall survival was 15.4 months. Overall survival after 1 year was 52 %. Three out of 11 deaths occurred in patients with extraperitoneal disease, with a median survival of 3.5 months (Fig. 3).

## Discussion

In patients with cancer confined to the peritoneal cavity, there is established pharmacokinetic evidence that intraperitoneal drug administration is advantageous.^20^ Intraperitoneal chemotherapy may improve survival of patients with gastric peritoneal metastasis.^21^ However, owing to the limited penetration of chemotherapy into tumor nodules, intraperitoneal chemotherapy may be best suited for small volume disease.^22^

For treating larger lesions, it has been proposed to perform a complete surgical cytoreduction prior to intraperitoneal chemotherapy.^23^ In gastric cancer, combining cytoreductive surgery with heated intraperitoneal chemotherapy (HIPEC) allowed long-term survival in a selected group of patients with limited peritoneal disease.^24^ However, this procedure has significant complications with a 30-day mortality of 5 %.^25^ Moreover, pharmacokinetic problems such as poor tumor penetration and incomplete irrigation of serosal surfaces limit the effectiveness of HIPEC.^26^ Thus, the benefit of adding HIPEC to cytoreductive surgery might be only marginal^26^ and its role in gastric cancer remains a matter of debate,^27^ in particular for signet-ring histology.

Prior work has predicted that innovative concepts overcoming pharmacologic limitations of intraperitoneal chemotherapy could improve, perhaps dramatically, its efficacy.^26^ While PIPAC remains in its infancy, the pharmacological superiority of this drug delivery system over systemic delivery and conventional intraperitoneal chemotherapy for treating peritoneal metastasis is already clear,^11^–^13^ inducing high response rates with low adverse events.^15^,^16^

We now report on the first results obtained with PIPAC with low-dose cisplatin and doxorubicin in patients with chemotherapy-resistant gastric peritoneal metastasis. Objective tumor response was documented in half of the patients after PIPAC, including complete histological regression in 6 patients. These findings confirm and extend those obtained in ovarian (reviewed in^28^) and colorectal^29^ cancer. The present results obtained in gastric cancer deliver further evidence suggesting that PIPAC can induce regression of platinum-resistant peritoneal metastasis in several cancer types and might meet the clinical need for new and better therapies for a fatal cancer.

However, in the present study, the 17 patients able to undergo more than one PIPAC procedure were indeed the patients who remained alive, presumably because their tumors responded to the first PIPAC. This is a selection bias, and it is therefore not a surprise that the majority of these patients had a favorable pathologic response. Thus, the response rate above should not be extrapolated to all treated patients, and these numbers have now to be confirmed in a prospective comparative study.

Intraperitoneal chemotherapy is hampered by dose-dependant, local toxicity. Local toxicity of PIPAC was acceptable, in spite of the high tissue drug concentration and of repeated delivery. No patient developed bowel perforation, and no severe gastrointestinal symptoms were registered. These results are in accordance with those reported in ovarian cancer, where gastrointestinal symptoms even improved slightly under PIPAC therapy.^16^

The systemic inflammatory response to the chemical peritonitis following intraperitoneal chemotherapy caused few general symptoms. In contrast to systemic chemotherapy, we did not observe typical side effects such as alopecia, neurotoxicity, cardiac toxicity, and myelosuppression. In accordance with previous observations,^14^–^16^,^29^ no significant renal toxicity was documented. This appears reasonable bearing in mind the 90 % dose reduction as compared to systemic chemotherapy. However, PIPAC induced transient low-grade liver toxicity in a quarter of patients, as reported earlier in ovarian cancer.^16^

Survival data are encouraging with a median survival of 15.4 months after first PIPAC application, confirming previous observations in ovarian peritoneal metastasis with a 1-year survival of 50 % in the third-line situation,^16^ and in colorectal cancer with a median survival of 15.7 months in the salvage situation.^29^ As an exception, gastric cancer patients with synchronous malignant pleural effusion did not benefit from PIPAC. Two patients with terminal disease and reduced condition (ECOG 3 and 4) died in the hospital after the procedure, suggesting that PIPAC is not helpful anymore in end-stage disease..

At this stage, it is not possible to define indications and contraindications for PIPAC. However, based on our preliminary experience, following observations are possible that might help to define inclusion and exclusion criteria for future studies: Since PIPAC can overcome platinum resistance, at least in some patients, it is expected to become a component of therapy of peritoneal metastasis. Time window for PIPAC appears to open when tumor nodes become resistant to platinum-based chemotherapy and to close when therapy-resistant ascites or bowel obstruction develops or when general condition deteriorates beyond ECOG 2. PIPAC might be indicated for older patients^30^ and for patients who experienced severe side effects from previous systemic chemotherapy, including chronic renal failure and cardiac toxicity. PIPAC has probably no clinical benefit in patients with malignant pleural effusion of gastric origin.

Our results provide first evidence that low-dose PIPAC therapy might be effective in treating patients with recurrent, platinum-resistant gastric peritoneal metastasis, including the aggressive signet-ring histology. PIPAC is well tolerated, a decisive characteristic in this palliative setting. However, these results are preliminary and should be interpreted conservatively. Due to the framework conditions (off-label use program without predefined inclusion and exclusion criteria), the patient cohort was heterogeneous. Thus, these results cannot be extrapolated to all patients with gastric peritoneal metastasis.

Future work includes a prospective, regulatory phase-2 clinical trial (NCT01854255) in the salvage situation. Further research is needed to determine if PIPAC might be indicated as a neo-adjuvant, additive, or adjuvant therapy in gastric cancer.

## Electronic supplementary material

Suppl. Material 1 42 y.o. male patient with progressive peritoneal carcinomatosis from signet-ring cell GC after 2 lines SC (ECF and FLOT). Partial pathologic response (PR) after 3 PIPAC and 4.5 months follow-up. Secondary limited small bowel resection was performed in order to remove remaining macroscopic disease. Macroscopy of the surgical specimen. a) overview of the scarred centimetric peritoneal carcinomatosis node (1,8 × 1,3 × 0,4 cm), b) mucosal ulceration, histology showed no tumor within the mucosa; c) perpendicular section through the bowel wall, showing transmural tumor scarring over several millimeters: d) narrow view: arrows show the direction of penetration of aerosolized chemotherapy. Histology revealed transmural fibrosis with 30 % vital tumor cells (major response) down to the submucosa, and the ulceration was healed suggesting penetration of chemotherapy throughout the bowel wall. Patient was alive 386 days after PIPAC #1. GC: gastric cancer. PC: peritoneal carcinomatosis. PCI = Peritoneal Cancer Index (JPEG 129 kb)

High resolution image (TIFF 34386 kb)

## Abbreviations

ASA: American Society of Anesthesiologists score
CC: Sugarbaker’s Index of Completeness of Cytoreduction
CR: Complete microscopic remission
CRS: Cytoreductive surgery
CTCAE: Common Terminology Criteria for Adverse Events
ECOG: Eastern Cooperative Oncology Group
GC: Gastric cancer
HIPEC: Hyperthermic intraperitoneal chemotherapy
IPC: Intraperitoneal chemotherapy
KI: Karnofsky index
PM: Peritoneal metastasis
PCI: Sugarbaker’s Peritoneal Cancer Index
PD: Progression of disease
PIPAC: Pressurized intraperitoneal aerosol chemotherapy
PR: Partial microscopic remission
SD: Stable disease
ULN: Upper limit of normal
WNL: Within normal limits
