# Supplementary figures and images for: Pressurized Intraperitoneal Aerosol Chemotherapy (PIPAC) with Low-Dose Cisplatin and Doxorubicin in Gastric Peritoneal Metastasis
Source: J Gastrointest Surg. 2015 Oct 28;20:367–73. doi: 10.1007/s11605-015-2995-9 (PMC4722080; doi:10.1007/s11605-015-2995-9)

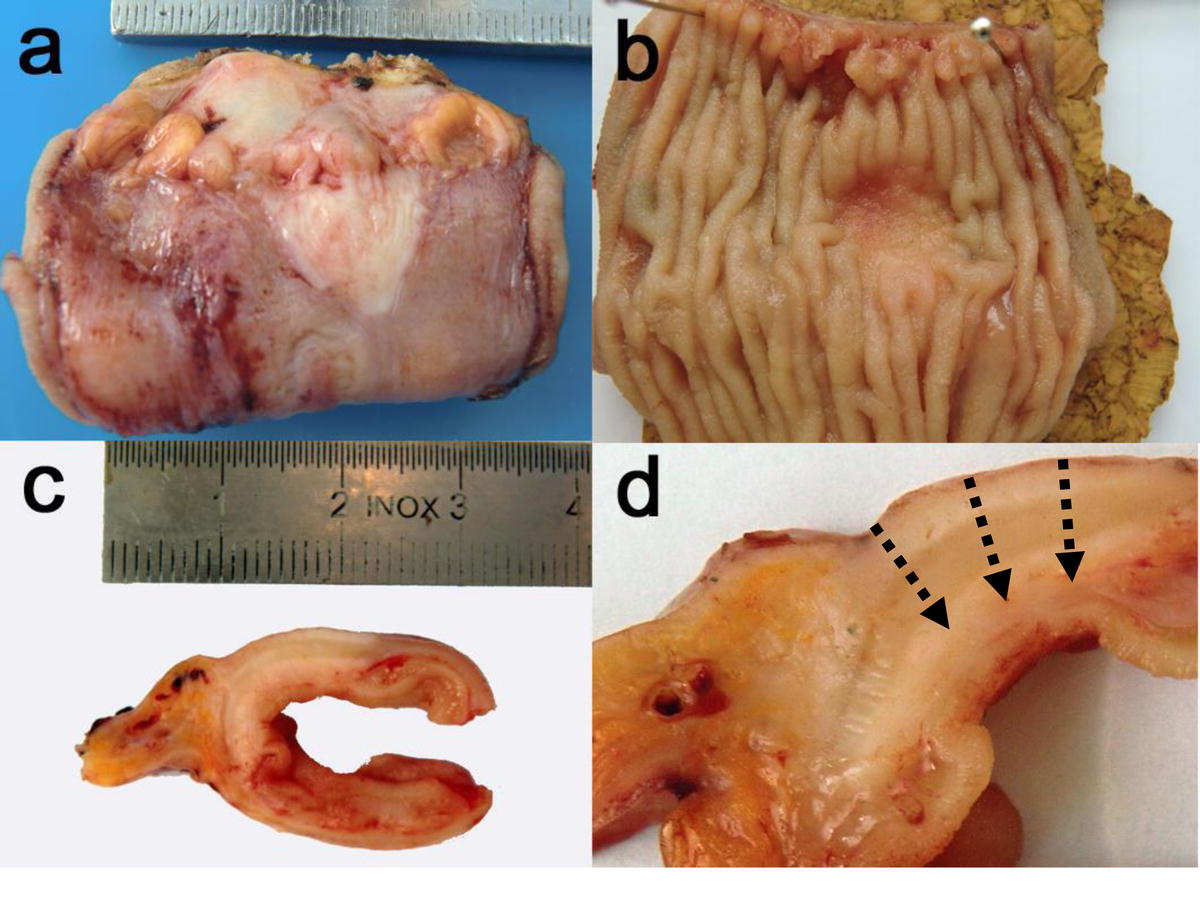

Supplement: Supplementary file 1 — 42 y.o. male patient with progressive peritoneal carcinomatosis from signet-ring cell GC after 2 lines SC (ECF and FLOT). Partial pathologic response (PR) after 3 PIPAC and 4.5 months follow-up. Secondary limited small bowel resection was performed in order to remove remaining macroscopic disease. Macroscopy of the surgical specimen. a) overview of the scarred centimetric peritoneal carcinomatosis node (1,8 × 1,3 × 0,4 cm), b) mucosal ulceration, histology showed no tumor within the mucosa; c) perpendicular section through the bowel wall, showing transmural tumor scarring over several millimeters: d) narrow view: arrows show the direction of penetration of aerosolized chemotherapy. Histology revealed transmural fibrosis with 30 % vital tumor cells (major response) down to the submucosa, and the ulceration was healed suggesting penetration of chemotherapy throughout the bowel wall. Patient was alive 386 days after PIPAC #1. GC: gastric cancer. PC: peritoneal carcinomatosis. PCI = Peritoneal Cancer Index (JPEG 129 kb) [file 11605_2015_2995_Fig4_ESM.jpg]

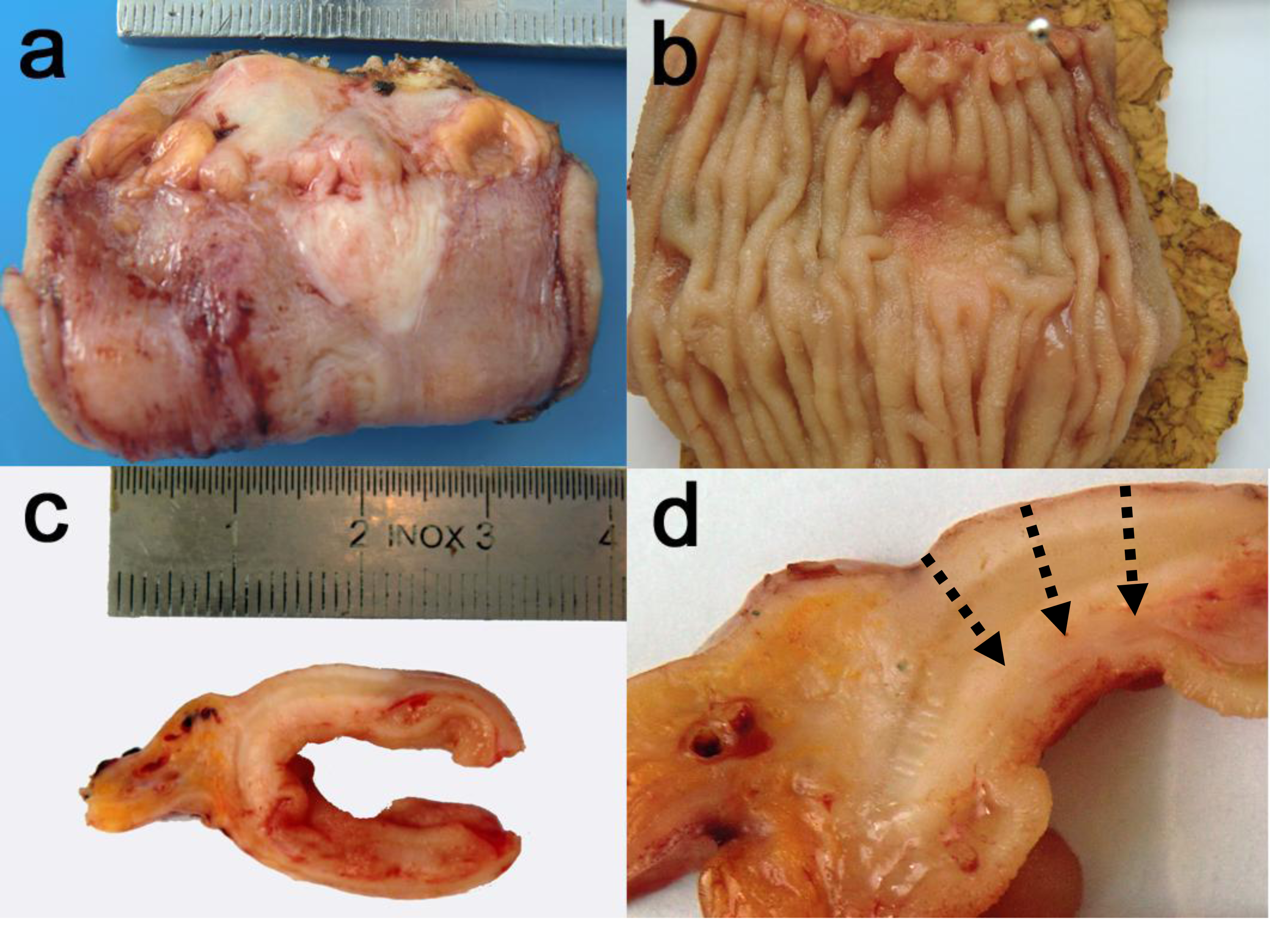

Supplement: Supplementary file 2 — High resolution image (TIFF 34386 kb) [file 11605_2015_2995_MOESM1_ESM.tif]
